# Ameloblastic Carcinoma with Calcification: A Rare Case Report in the Mandible and Literature Review

**DOI:** 10.1155/2020/4216489

**Published:** 2020-10-13

**Authors:** Neda Kardouni Khoozestani, Farzaneh Mosavat, Mohammad Shirkhoda, Ramtin Azar

**Affiliations:** ^1^Departments of Oral and Maxillofacial Pathology, Cancer Institute, Tehran University of Medical Sciences, Tehran, Iran; ^2^Oral and Maxillofacial Radiology, School of Dentistry, Tehran University of Medical Sciences, Tehran, Iran; ^3^Department of Onco-Surgery, Cancer Institute, Tehran University of Medical Sciences, Tehran, Iran

## Abstract

Ameloblastic carcinoma (AC) is a scarce malignant tumor which is more prevalent in the mandible than the maxilla. It occurs in a wide range of age groups, and there is a sex predilection in males. AC shows specific microscopic features and requires more aggressive surgical treatment plans in comparison with conventional ameloblastoma. Radiographically, AC resembles ameloblastoma except that it rarely represents focal mineralized materials, seemingly reflecting dystrophic calcification. This characteristic is uncommon in typical ameloblastomas, and only few cases reported with such opacities and mineralized materials. Due to this rare radiographic and microscopic presentation, an accurate diagnosis could be challenging, and pathologists should consider a combination of benign and malignant odontogenic tumors occurring in jaws.

## 1. Introduction

Odontogenic malignancies are rare lesions which comprise one percent of all cysts and tumors happening in the jaws arising from tissues associated with odontogenic epithelium [1–3]. AC is considered a rare entity that shows the histopathological characteristics of ameloblastoma with histological atypia regardless of metastasis occurring or not [2].

Considering the rarity and diverse nomenclature of this lesion, AC has been a topic of diagnostic challenges for a long period of time [3]. The most common sign of AC is swelling; nevertheless, symptoms such as trismus, pain, dysphonia, and rapid growth also may occur [1, 4, 5]. However, in 2017, the World Health Organization announced that there seems to be little justification for dividing such a rare tumor into smaller subsets; therefore, it continues simply as a single entity (ameloblastic carcinoma) [6].

Radiographically, AC looks like ameloblastoma except that it occasionally shows focal radiopaque materials; apparently, it reflects dystrophic calcification itself. This trend does not often appear in typical ameloblastomas [4]. The literature was searched according to the combination of the following terms on PubMed: ((calcifi∗) OR minerali∗) AND (ameloblastoma) [Mesh].

We documented a case of recurrent AC with calcification in a 40-year-old woman who underwent surgery and radiotherapy.

## 2. Case Report

A 40-year-old woman referred to Imam Khomeini Cancer Institute was complaining of intense lower facial pain and progressive swelling for the period of the last six months. Although she has not had a considerable problem in her past medical history, she has also given us a history of her left second premolar and first molar teeth root canal therapy in which she had not noticed any change in pain and swollen area size. She had also undergone incisional biopsy in another health care center with pathologic results designated as a “malignant Pindborg tumor.”

On clinical examination, diffuse swelling causing lower lip tenderness and paresthesia with extension to the left posterior part of the mandible was detected. On examination, a mass that measures approximately 4 cm with a rubbery consistency was found. The overlying skin tissue was intact; however, oral mucosa was ulcerated. No palpable subcutaneous cervical lymph node was found. There was no difficulty in swallowing, dysphagia, trismus, or dysphonia.

**Figure 1:**
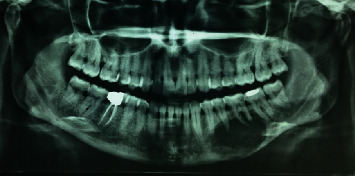
Panoramic radiograph showing a radiolucent lesion in the left mandible body containing small radiopaque foci, especially in the lower parts.

**Figure 2:**
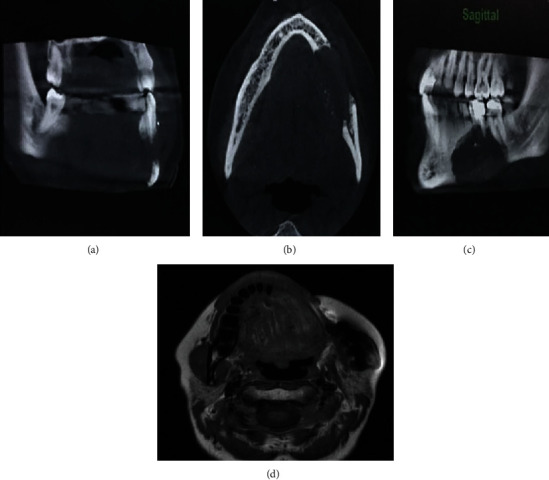
(a–c) Coronal, axial, and sagittal CBCT views showing severe buccal and lingual cortical plate perforation and IAC involvement by the tumor. The small radiopaque foci marked with arrows (b, c). (d) T1-weighted MRI showing left hemimandibular excision with its ipsilateral masseter muscle resection from the left lateral incisor area.

**Figure 3:**
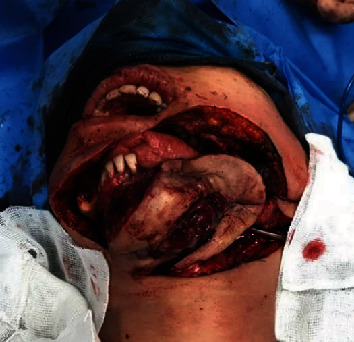
Patient surgical photography showing hemimandibulectomy which provides safe margin removal.

On panoramic and cone-beam computed tomography (CBCT), the lesion with a well-defined border and internal focal radiopacities was seen that extended from the periapical area of the mandibular left canine to the mandibular left second molar in an anteroposterior dimension and from the alveolar crest to the inferior border of the mandible in a superoinferior aspect (Figure 1). Buccal and lingual cortical plate perforation was also detected. The inferior alveolar canal (IAC) was involved by the lesion, which confirmed the patient's history of pain and paresthesia (Figure 2). After multidisciplinary evaluations and elucidation of the lesion destructive nature in radiographs, left hemimandibulectomy was performed for the patient (Figure 3).

**Figure 4:**
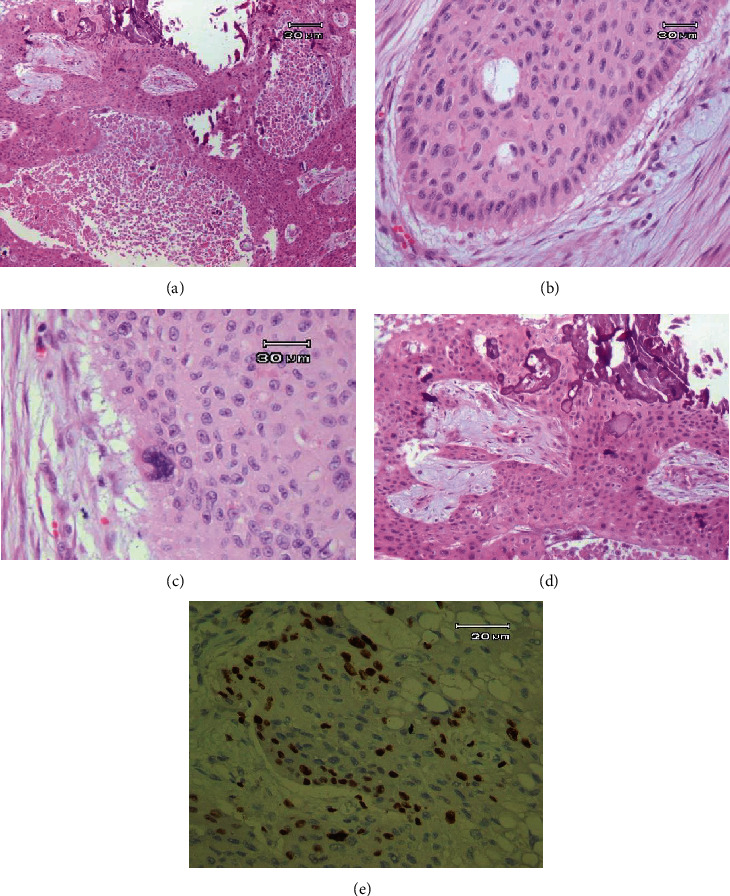
Histopathological features of ameloblastic carcinoma. (a) Ameloblastic carcinoma depicting the extensive area of calcified materials at the center of islands as well as in the intracellular areas (hematoxylin and eosin, ×40). (b) Follicles and islands with marked palisaded tall columnar cells at the periphery (hematoxylin and eosin, ×400). (c) Tumoral cells with pleomorphism, cellular atypia, loss of peripheral palisading, nuclear polarity, and presence of tumoral giant cell (hematoxylin and eosin, ×400). (d) Amorphous eosinophilic intercellular secretion which undergoes calcifications in some foci (hematoxylin and eosin, ×100). (e) Ki-67 immunostaining in ameloblastic carcinoma showed a significantly high Ki-67 labeling index especially at the periphery of the islands (×400).

Microscopic examinations showed odontogenic epithelial neoplasm composed of sheet and island of epithelial cells with malignant features including cellular pleomorphism, hyperchromatism, few atypical mitotic figures, central necrosis, and perineural invasion in a collagenized stroma. At the periphery of the islands, tall columnar cells resembling ameloblast cells with nuclear palisading, reverse polarity, and subnuclear vacuolization are evident. The particular feature of this tumor was the presence of intercellular eosinophilic amorphous materials in extensive areas that had been transformed into mineralized materials in some parts. The lesion was superficially covered by ulcerated stratified squamous epithelium (Figure 4). Besides, Ki-67 immunostaining was performed, and its expression was noticeable (around 45% in HPF), especially at the peripheral cells and occasionally at the central cells of the tumor islands (Figure 4(e)).

## 3. Discussion

Odontogenic malignancies are rare lesions which are composed of one percent of all cysts and tumors appearing in the jaws emanating from tissue associated with odontogenic epithelium [1, 5, 7, 8]. For the first time in 1984, Shafer described the ameloblastic carcinoma as ameloblastoma that shows some extent of cellular pleomorphism and necrosis [4].

Varying signaling pathways which take part in the progression of ameloblastomas have been recognized highlighting the MAPK signaling pathway with its predominant B-RAF proto-oncogene serine/threonine kinase (BRAF) V600E mutation [9]. Since the presence of B-RAF proto-oncogene serine/threonine kinase (BRAF) V600E mutation in ameloblastoma and some cases of ameloblastic carcinoma has been reported, which may be involved in its behavior, targeted therapies have been used as an alternative in the case of resistance or recurrent ameloblastoma and ameloblastic carcinoma [9].

Based on a systematic literature review which has been carried out by Deng et al. from January 2000 to July 2018 and our literature review, AC presents a higher male prevalence when compared to females. The most involved site in the jaw is the posterior area of the mandible with a 64% occurrence rate [10]. AC can occur in a wide age range; however, the average age of 45 years is considered in the literature. The most usual symptom of AC is swelling (68%), although AC may be accompanied by pain, fast growth, difficulty in jaw functions, and dysphonia [4, 10]. Our case was conducted more or less in the same age group which revealed no difficulty in swallowing, dysphagia, trismus, and dysphonia.

Based on radiographic view, differential diagnosis of posterior mandibular radiolucency includes odontogenic keratocyst, myxoma, Pindborg tumor, ameloblastoma, and less commonly intraosseous malignancies or metastatic lesions [10, 11]. The radiographic findings do not help the diagnosis, although the destructive nature of the presented case in radiographs is more in favor of malignant neoplasms, including ameloblastic carcinoma as well.

Differentiation between ameloblastic carcinoma and classic ameloblastoma may be impossible based on the radiological aspect solely. Both of the lesions demonstrate a bony hard expansive mass with teeth displacement, loss of lamina dura, root resorption, and occasionally teeth loosening [12, 13]. Like classic ameloblastoma, AC is mostly found in the premolar and molar regions of the mandible. Both of them typically have a well-defined and corticated border, although sometimes a scalloped border can be observed [14]. AC similar to ameloblastoma is unilocular or more commonly multilocular radiolucent. Most of the septa in AC are course and thick, which is seen also in classic ameloblastoma and can lead to a honeycomb- or soap bubble-like pattern in radiographic appearances. However, AC typically shows more features such as dystrophic calcifications and mixed solid and cystic regions [12–15].

As mentioned in the radiographic features of the discussed case, there were severe buccal and lingual cortical plate perforation, root blunting of a mandibular first premolar, and root resorption in a mandibular first molar as well as the appearance of dystrophic calcifications. The notable radiologic feature of this patient was the presence of multiple small internal radiopacities throughout the lesion. Based on our literature review, two cases of AC have been represented with calcification [10, 16]. Similarly, a systematic literature review published in 2019 which had been done by Deng et al. from 2000 to 2018 strengthens our results [10, 16].

On occasion, calcification may be a result of intensive apoptotic activity, and the final differentiation of keratinocytes is taught out to be a variant of the apoptotic process [7, 10, 17, 18]. Histologically, the main differential diagnosis of AC with cellular atypia, necrosis, and such extensive calcified materials could carry the malignant calcifying epithelial odontogenic tumor, squamous odontogenic tumor with malignant features, and metastatic carcinoma from the unknown primary origin. Due to observation of some calcified foci, our case mistakenly had been reported as a malignant Pindborg tumor. Regarding the existence of palisaded columnar cells at the periphery of neoplastic islands, the malignant Pindborg tumor which was formed from polygonal to squamoid cells with atypical cytologic features can be ruled out [3, 4, 19]. The amyloid stain negativity of eosinophilic amorphous intercellular materials has also verified the diagnosis. The next differential diagnosis which was ruled out was the squamous odontogenic tumor with malignant features. Rare entity neoplasm does not show palisaded tall columnar cells at the periphery of islands; moreover, it lacks stellate reticulum-like cells in the island's center and, the last but not least, lacks intercellular secretions in both the WHO and simple types of this neoplasm [19, 20]. The third pathological differential diagnosis was excluded due to the patient's clinical history and complete metastasis workup that no other origin was detected.

Looking at the microscopic features of this case in a different angle reveals that the tangible absence of stellate reticulum-like cells in the center of islands, trabeculae, and sheets of the neoplastic cells is highly in favor of AC. In this regard, Angiero et al. pointed out that the presence of nests, islands, sheets, or trabeculae of reverse polarized epithelium with scarce or no existence of stellate reticulum-like areas can be considered a diagnostic clue for the pathologist to appraise AC in differential diagnosis [21]. The Ki-67 labeling index shows a statistically significant difference between ameloblastoma and ameloblastic carcinoma, which ranges from 11.87% to 53.29% in the case of the latter [22]. Ameloblastic carcinoma by itself is a rare lesion, and AC with calcification is much more uncommon, and according to the literature review, only a few cases have been reported so far [4, 10]. Loyola et al. noted an infrequent but interesting presence of calcification in the vicinity of malignant odontogenic epithelium in the AC, which in turn can complicate the diagnosis [23]. Fitzpatrick et al. reported a case of ameloblastic carcinoma with features of ghost cell odontogenic carcinoma in the anterior area of the maxilla in a 37-year-old man which revealed multiple calcifications histologically [24]. Casaroto et al. reviewed cases of ameloblastic carcinoma and found that 12.5% of cases exhibited calcification and three (12.5%) had rosette-like structures [25]. Although ghost cells, keratin production, calcifications, and rosette-like structures were rare in AC cases, these features should also be carefully evaluated when present in this tumor [25]. Another study analyzed 14 cases of ameloblastoma, one of which showed calcification. It can be concluded that since calcification in ameloblastoma is rare, the presence of these calcifications must be carefully evaluated [26].

There are different methods to tackle AC, including surgical resection with a safe margin with or without cervical lymph node removal and surgical removal with or without chemotherapy. Having a safe margin can reduce the rate of recurrence to less than 15% [10, 27]. Another treatment modality that had been reported in the literature review for these patients is surgical resection with neck dissection with or without radiotherapy (29.4% recurrence) [10, 27]. The lung is the most prevalent region (9%) for distant metastasis according to the literature review [10, 27].

Our case underwent hemimandibulectomy and radiotherapy. We followed the patient for four years after the initial diagnosis of AC. She is still alive but unfortunately suffering from multiple lung metastases and poor general health quality.

## 4. Conclusion

Dystrophic calcifications are rare findings in ACs. However, the presence of such materials as an occasional feature can be considered a diagnostic point for pathologists. It seems that more cases are needed to be studied to understand how these calcifications could relate to the patient's outcomes.
